# Social Brains in Context: Lesions Targeted to the Song Control System in Female Cowbirds Affect Their Social Network

**DOI:** 10.1371/journal.pone.0063239

**Published:** 2013-05-01

**Authors:** Sarah E. Maguire, Marc F. Schmidt, David J. White

**Affiliations:** 1 Department of Neuroscience, University of Pennsylvania School of Medicine, Philadelphia, Pennsylvania, United States of America; 2 Department of Biology, University of Pennsylvania, Philadelphia, Pennsylvania, United States of America; 3 Department of Psychology, Wilfrid Laurier University, Waterloo, Ontario, Canada; Cajal Institute, Consejo Superior de Investigaciones Científicas, Spain

## Abstract

Social experiences can organize physiological, neural, and reproductive function, but there are few experimental preparations that allow one to study the effect individuals have in structuring their social environment. We examined the connections between mechanisms underlying individual behavior and social dynamics in flocks of brown-headed cowbirds (*Molothrus ater*). We conducted targeted inactivations of the neural song control system in female subjects. Playback tests revealed that the lesions affected females' song preferences: lesioned females were no longer selective for high quality conspecific song. Instead, they reacted to all cowbird songs vigorously. When lesioned females were introduced into mixed-sex captive flocks, they were less likely to form strong pair-bonds, and they no longer showed preferences for dominant males. This in turn created a cascade of effects through the groups. Social network analyses showed that the introduction of the lesioned females created instabilities in the social structure: males in the groups changed their dominance status and their courtship patterns, and even the competitive behavior of other female group-mates was affected. These results reveal that inactivation of the song control system in female cowbirds not only affects individual behavior, but also exerts widespread effects on the stability of the entire social system.

## Introduction

Songbirds offer a unique opportunity to study social behavior because much is known about the mechanistic control and evolutionary function of their central social trait, their song. From a proximate perspective, the neural architecture underlying song production has been clearly outlined [1], [2]. From an evolutionary perspective, observations in the wild have revealed how song production and song perception relate to reproductive success. Males use song to compete with other males and to court females. Females have preferences for songs, which allow them to mate with males who have high heritable fitness [3]. While birdsong research has provided valuable tools and techniques for studying song, the song signal itself, however, is only one component of a communication network involving many individuals and the relationship between sociality and song can be quite complex [4], [5], [6].

Cowbirds provide a good study system for exploring how individual characteristics interact to create a social network because much is known about their song signal as well as their group dynamics. Male cowbirds use song to establish dominance relationships with males and to court females. Dominant males use song to suppress subordinates' activity and thus gain more access to females [7]. Males court females by singing to them repeatedly. Effective courtship takes place at close distance and can be paired with visual displays. In the breeding season, male courtship becomes focused on individual females and pair-bonds form. Males remain close to their pair-mate, sing to them repeatedly, and defend them from singing overtures of other males. Thus females are courted primarily by their pair-mate. Past work has indicated that males who are most successful in mating, selectively court and defend their female such that their songs account for over 85 percent of all courtship song their female will receive in a breeding season [8], [9] and in many cases this selectivity can be much higher. While most research has focused on the singing behavior of males during the breeding season, non-singing females are also active – but much more subtle – participants in pair-bonding [5]. In order for males to elicit a female's copulation solicitation display, they must sing to the female at a close distance (typically within 30 cm). Females can exercise their choice of males by regulating their social distance, providing or removing opportunities for males to sing to them at close distances. Females also use a ‘chatter’ vocalization to attract males. Females in pair-bonds often chatter immediately in response to their pair-mate's songs, which may serve to maintain the pair-bond [10]. Additionally, females compete with other females for access to mates [10]. Taken together, features related to song production and perception combine in complex ways to produce the social interactions necessary for effective breeding.

The challenge inherent in understanding the mechanistic basis of social behavior comes in studying the feedback loop of effects that exists in all social systems: the behaviors of individuals interact to create the dynamics of the group, but those group dynamics also influence the individuals' behaviors. In the current investigation, we created an experimental probe into this feedback loop. We modified one aspect of the behaviour of a subset of individuals and then used those individuals as probes into the structure and malleability of their social groups. We focused on modifying females' song preferences because of past work indicating that females' preferences can play subtle, but important roles in affecting male behavior. To modify song preferences, we inactivated the song control system in females through targeted lesions of the nucleus HVC – an important nucleus for female selectivity for conspecific song [11], [12], [13], [14], [15], [16], [17], as well as some of the auditory structures surrounding HVC [18], [19], as this has been shown to be the most effective approach for eliminating selectivity to conspecific song.

We conducted two experiments using females with HVC targeted lesions. First, using playback tests we examined the selectivity that lesioned females displayed in producing copulation solicitation displays to variants of conspecific and heterospecific song. In the second experiment, we introduced lesioned females into mixed-sex flocks breeding in captive aviaries to examine how changes to the behavior of a subset of individuals could influence the function of the groups and the behaviour of the group-mates.

## Materials and Methods

### Ethics Statement

The Institutional Animal Care and Use Committee (IACUC) at the University of Pennsylvania specifically approved this study (protocol numbers 704383 and 800439). All surgeries were carried out under ketamine/xylazine, and all efforts were made to minimize discomfort. All birds used in this study were wild-caught in Montgomery County, PA, USA under appropriate state and federal trapping permits.

### Subjects and housing

12 adult male and 32 adult female brown-headed cowbirds served as subjects in the experiment. Upon capture, we placed unique color combinations of leg bands on each bird to permit individual identification. All birds were housed in large (18.3×6.1×4 m) outdoor aviaries. Aviaries contained grass, trees, shrubs, feeding shelters, and perches. Birds had access to a mix of millet and canary seed plus a modified Bronx zoo diet for omnivorous birds and vitamin-treated water *ad libitum*.

### Surgical and Anatomical Methods

Chemical lesions were targeted to HVC as previously published [11], [20]. Briefly, birds were first given an intramuscular injection of 5 mg/kg diazepam followed 20 minutes later by an injection of ketamine/xylazine (35/7 mg/kg). Birds were then placed in a stereotaxic apparatus that allowed their heads to be tilted to a 45° angle. A portion of the outer skull layer overlaying the right and left HVC was removed and HVC was targeted using stereotaxic coordinates relative to the bifurcation of the central sinus (A–P: 0, M–L: ±3 mm, D–V: −0.7 mm, personal communication with Roderick Suthers). With the aid of a surgical microscope, glass pipettes were mounted to a nanoject and filled with either the neurotoxin Ibotenic acid (Sigma; 0.66% ibotenic acid; 10 mg in 1.52 ml in 0.4 M phosphate buffer, final pH 7.6) or the buffer alone. Spontaneous bursting patterns that are characteristic of HVC were used to confirm the location of the nucleus. Lesions were made by slowly injecting up to 0.4 µl of ibotenic acid (lesion group) or phosphate buffer (sham-lesion group) into the right and left HVC. After each injection, the electrode was left in place for at least 5 minutes to prevent spreading up the electrode track. Birds were then allowed to recover for at least four days after the surgery. Given the small size of HVC in female cowbirds, these procedures always lesioned all of HVC as well as some of the tissue surrounding HVC.

Six weeks after surgery, four control birds and four experimental birds were anesthetized with Nembutal (0.2 ml of 50 mg/ml; Abbott Laboratories, Chicago, IL) and perfused with 0.9% saline followed by 4% paraformaldehyde. Brains were then removed and further postfixed in 4% formaldehyde and cryoprotected using 30% sucrose. Brains were cut on a freezing microtome into 40 µm parasagittal sections, which were then mounted and Nissl stained. The success of the lesion was confirmed visually for each of the perfused birds. Lesioned birds sustained damage to the injection area while sham-lesioned birds did not.

### Experiment 1: Playback Procedures

Experiments to evaluate the effect of lesions on female song preference were performed on a set of 8 lesioned and 8 sham-lesioned females starting May 8, 2011. We conducted surgeries as described above and, after recovery, placed the females into 1 m^3^ sound attenuation chambers. Based on previously published techniques [8], we assessed the rates of copulation solicitation displays produced by females in the two conditions to broadcasts of recordings of male songs. We selected 10 recordings of crystallized, mature male cowbird song recorded in aviaries in May 2008 using Sennheiser RF condenser microphones recorded on a Marantz solid state digital recorder. Songs were played from a computer through an LG XDSS amplifier to Bose 161 speakers located in each sound-attenuating chamber. The sound pressure levels of the songs were 85±2 dB (a weighted impulse reading at 0.8 m from the speaker as recorded by a B&K 2209 sound pressure meter). Signal to noise ratios, measuring peak noise to peak signal, did not differ among songs.

During playback experiments, we housed females in pairs in the chambers because it serves to reduce females' stress and has been shown to have no influence on females' responses to playback [21], [22], [23]. Nevertheless, we only paired females from the same surgical condition together. We played 6 songs per day to females across 10 days. Each song was approximately one second long and song playbacks were separated by 90 min. We alternated the order in which we played songs with each song presented six times over the course of the experiment. We scored a positive response if a female adopted a copulation solicitation display within one second from the onset of the song. The display is similar to the lordosis display in mammals, characterized by the arching of the back, and the spreading of the wing and cloacal feathers. To calculate each song's quality (its effectiveness at eliciting a copulatory posture), we computed the mean number of copulation solicitation displays each female gave for each song and averaged over all females in each condition. The selected songs had been used in playback tests in May 2010 with 16 unmanipulated wild-caught females using the same procedures as described above. We thus had information about each song's quality from these females and could correlate relative preferences from the unmanipulated females with the subjects in the current experiment.

In the second playback test, we compared 6 new local dialect songs to 6 songs recorded from heterospecifics. These recordings were from Cornell University's Laboratory of Ornithology. We played songs from a variety of species, many of which are local to the area. We used songs from 6 species: American redstart (*Setophaga ruticilla*), Bell's vireo (*Vireo bellii*), yellow warbler (*Dendroica petechia*), song sparrow (*Melospiza melodia*), red-winged blackbird (*Agelaius phoeniceus*), and barn swallow (*Hirundo rustica*). Signal to noise ratios and peak amplitude were similar to the cowbird songs. We only played songs in this playback session 4 times each because the effects were so dramatic. One sham-lesioned female failed to produce any displays throughout testing and one lesioned female died during testing. Their results were removed from analysis.

### Experiment 2: Aviary Introductions

**Figure 1:**
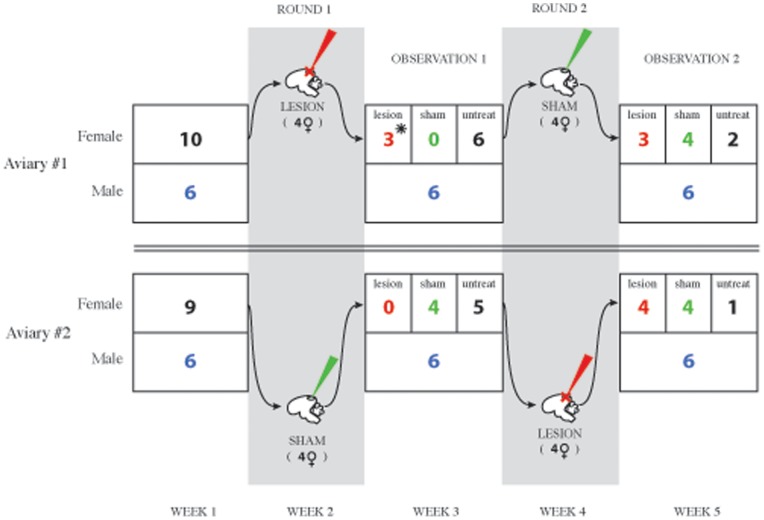
Timing of manipulations in experiment 2. *1 Lesioned female died in surgery in round 1 aviary 1.

Birds used for this experiment were captured between April 5 and April 17, 2010 and kept in same-sex holding aviaries. The time course of experiment 2 is shown in Figure 1. On April 20, 2010, we randomly assigned 6 males and 10 females to two aviaries. Birds remained housed in these conditions until the first removals for surgical treatments occurred (May 10). This corresponds to the approximate beginning of the cowbird breeding season. We collected behavioral data on the birds in these conditions daily (see below for details of behavioral data collection). One female died soon after the aviaries were set up (in aviary 2) and was removed from the analysis. Three females (2 in aviary 1 and 1 in aviary 2) engaged in no interactions with males or with other females and thus were not used as subjects for lesioned or sham lesions. We categorized these females as members of the ‘untreat’ group in Figure 1. These females remained in the aviaries but their data were not used for the experiment. On May 10 (ROUND 1), we removed four randomly selected females from each of the aviaries. We performed HVC lesions to the four females from Aviary 1 (one died during surgery, represented with an asterisk in Figure 1) and sham lesions to the females from Aviary 2. After seven days, the lesioned females were re-introduced late in the afternoon into Aviary 1 and sham-lesioned females were re-introduced at the same time into Aviary 2. Starting at 600 h the following day, we took behavioral observations on all birds. After one week of data collection, we conducted another round of removals (ROUND 2), surgeries, and reintroductions. This time introductions were counterbalanced such that sham-lesioned females were introduced into Aviary 1 and lesioned females were introduced into Aviary 2. This was followed by another week of behavioral data collection.

### Behavioral Censuses

We collected data on singing and social interactions in both aviaries. In each aviary we collected data for two hours per day between 600 h and 1000 h. Data collection involved all occasion sampling of singing behavior and reactions using previously published techniques [5]. Briefly, we noted patterns and amount of male vocalizations in 15-minute censuses. We recorded behavior in the aviaries using an automated speech recognition system, which allowed observers to speak codes of singing interactions into wireless microphones [24]. These data were then automatically checked for errors, summarized and analyzed in real time by programmable databases. Interobserver reliability was high using voice recognition for song censuses (songs per male; r = 0.98, p<0.001; [24]). Within each census, we noted the individual who sang, whether it was directed to another bird or was undirected. Directed songs could be male-directed or female-directed. The criteria for scoring a directed song in either context was that a song had to be sung within 30 cm of another bird and the singer had to be oriented on axis between 0 and 30 degrees toward the other male or female. While birds are able to hear beyond this distance, songs that occur at close-distance are especially important for social interactions. For male-male singing, countersinging bouts only occur within this close distance (where males sing back and forth in quick succession). Countersinging is what escalates into antagonistic and dominance interactions, such as fights and pecks [5]. We measured all instances of these interactions. Courtship singing also must occur at close distance because song amplitude attenuates across distance very quickly and amplitude is important for eliciting females' copulation solicitation displays [25]. From past research, we have never observed a copulation solicited from a male who sang from more than 30 cm away.

### Data Analysis

The experimental design allowed for the comparison of the two groups at the exact same time window in the breeding season. This was done instead of comparing changes within-subjects across time because, based on past experience with cowbird breeding behavior using unmanipulated females, we have found that female reproductive condition and behavior can change dramatically across the timeframe of a breeding season [5], [10]. We were able to make some within-subject comparisons for behaviors that past research has suggested to be more stable. For example, female chatter typically remains consistent across breeding seasons, as does male dominance ranking, and social network structure [5], [10].

We used UCInet 6 (Analytic Technologies, Borgatti, Everett, Freeman, 2002) for analysis of social network structure on the directed singing networks assembled during the two observation sessions for each of the aviaries. For males, we compared how directed song networks metrics changed from the observation session when sham-lesioned females were introduced and when lesioned females were introduced. While there are a very large number of possible analyses that could be run, in order to limit family-wise error rates we selected metrics that past research have found to characterize groups in which individuals have high levels of reproductive success (described below).

Closeness: a normalized network centrality metric that measures geodesic distances of a male to all other nodes in the network. High closeness means each male has established singing interactions with many other males.

Ego networks can be calculated for each individual within the network. An individual's ego network refers to the connections that the individual has with others in the network. Ego network size refers to the number of others to whom the individual (‘ego’) directly sings. Thus in larger ego networks, males sing to more individuals within the group.

Finally, the number of weak components (or indirect connections) within an ego network was measured. Strong components refer to situations where all individuals within an ego network interact with one another. In contrast, weak components refer to individuals that are only linked together because of ego's directed singing interactions (i.e., they do not interact with one another, just with ego). Thus more weak components represent a more dispersed singing network where subgroups form and males sing predominantly with each other and not with the rest of the network.

We also tested indegree and outdegree centrality (measures of network activity that characterizes overall amounts of singing interactions occurring among males – either sung to others (outdegree) or songs received from others (indegree).

## Results

### Experiment 1: Lesions Eliminate Female Selectivity for Conspecific Song

**Figure 2:**
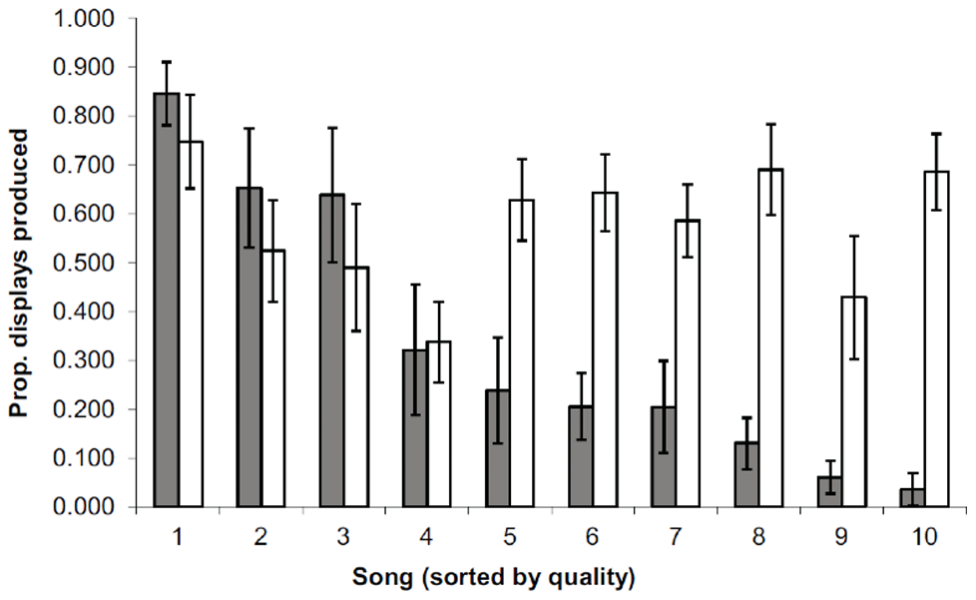
Lesions of the song control system cause females to lose song selectivity. Mean (±1 SEM) proportion of copulation solicitation displays produced by lesioned (open bars) and sham-lesioned (filled bars) females for playbacks of 10 local cowbird songs in experiment 1. Songs are ordered based on song quality as ranked by the displays of unmanipulated females in prior years of playback testing.

Sham-lesioned females displayed selectivity in responding to songs similar to the original wild-caught females (spearman r = .81, p<.005; Figure 2). The responses of lesioned females however, did not correlate with the sham-lesioned (r = −.07, NS) or original females (r = .15, NS). Instead, lesioned females produced robust copulatory responses to all of the songs in the playback set. However, selectivity was only lost for conspecific song. When we tested the females' preferences for conspecific versus heterospecific songs, we found no differences between lesioned and sham-lesioned females. Every female showed strong preferences for conspecific song (sham-lesioned: paired t-test, t(6) = 4.08, p<.01; lesioned: t(6) = 6.25, p<.001).

### Experiment 2: Female Selectivity Affects the Dynamics of the Social Network

The lesion manipulation had remarkably little influence on the overt, measurable aspects of the females' behaviors. Males sang to lesioned and sham-lesioned females at equal rates: lesioned females received 104.57±21.66 songs across the observation session, sham-lesioned females received 101.88±20.59 songs; t(13) = .09, p>.93). Females approached males at similar rates: lesioned females averaged 1±0.44 approach per female across the observation session, sham-lesioned females averaged 0.5±0.38 approaches; t(13) = 0.87, NS). They also engaged in similar rates of chatter production: sham-lesioned females tended to chatter more than lesioned females, but owing to the substantial variability across females, the difference was not significant: sham-lesioned females  = 92.75±40.92 chatters across the observation session, lesioned females  = 23.71±22.23 chatters; t(13) = 1.42, NS).

**Figure 3:**
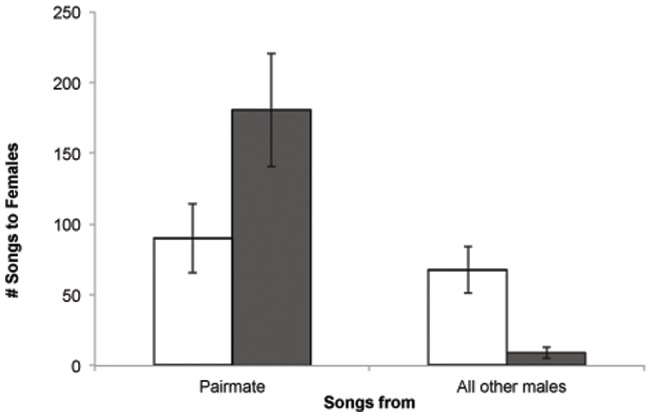
Lesioned females fail to pair-bond normally. Selectivity of directed singing by males to lesioned (open bars) and sham-lesioned (filled bars) females in experiment 2. Bars represent mean (±1 SEM) songs received from the pair-mate and from all other males for 7 lesioned and 8 sham-lesioned females.

These measures initially led us to believe that there were no effects of the lesions on the behavior of the females in the aviaries – females from the two conditions seemed to act remarkably similar. However, when we investigated the behaviour of the other birds in the groups, distinct differences emerged as a consequence of the female introductions: males courted females from the two conditions differently, meaning that the strength of the pair-bond was different for lesioned and sham-lesioned birds. Lesioned females received courtship song from many different males compared to sham-lesioned females (mean number of males singing more than one song to sham-lesioned females  = 1.75±.36, mean for lesioned females  = 3.86±.83, t(13) = 2.76, p<.02). In addition, sham-lesioned females received 97 (±0.64) percent of directed singing overtures from their pair-mate, whereas lesioned females only received 72.6 (±8.2) percent of song from their pair-mate, significantly lower than sham-lesioned females (t(6.07) = 2.98, p<.024; Figure 3). These differences in courtship patterns reveal that the strength of the pair-bond was weaker for lesioned females than for sham-lesioned birds.

Prior to this experiment, pair-bonding appeared to be primarily controlled by males through male-male competition, and mate guarding. Here however, a manipulation to females' song selectivity had a pronounced effect on the strength of pair-bonding. One potential behavioral mechanism by which females may have influenced males' singing selectivity was through their use of chatter. Females use chattering to attract the attention of males, especially that of their mates'. While both sham and lesioned females produced chatter, the two groups of females differed in the use of chatter. Sham females were more selective in chattering, allocating a higher proportion of their chatter in response to their pair-mates' song than lesioned females (t(9) = 2.43, p<.05).

Females also use chatter to attract the attention of males away from other females [10], which may induce female-female competition. To test this, we compared increases in chattering from the remaining females in the aviary after introducing lesioned females and sham-lesioned females. We found that the other females increased their chattering more when lesioned females were introduced than when sham-lesioned females were introduced (post lesion introductions, mean chatters  = 66.25±18.34, post sham introductions: 16.63±10.72, t(14) = 2.29, p<.04). Thus the introduction of lesioned females (and the changes in male courtship that ensued) incited more female-female competition over mates.

The introduction of lesioned females also affected males' dominance interactions. Since singing among males often leads one male to suppress the other male's singing, dominance could be measured by determining, for each male, how much he sang to another male compared to how much that other male sang to him. Dominant males have a higher ratio of “song sung” to “song received” than subordinate males [8]. Past work has revealed that this male-directed singing ratio correlates with other male dominance behaviors such as displacements and fights [8]. Furthermore, it relates to male reproductive success, and typically remains highly consistent within a group across time [5], [8]. To assess whether lesioned females changed male dominance relationships within the group, we compared the singing ratio for each male-pair immediately after the lesioned females were introduced and compared it to their respective singing ratio prior to the introductions. We found that there was a significantly greater change the average male singing ratio when lesioned females were added than when sham-lesioned females were added (mean change in singing ratio lesioned introduction  = 0.29±.05, sham-lesioned introduction  = 0.11±0.03; t(11) = 3.53, p<.005). This indicates that lesioned females caused the dynamics of male-male relationships in the aviary to change more than when sham-lesioned birds were added.

There are multiple ways dominance ranks among male pairs could change and influence the singing ratio. We tested whether the change in singing ratios were a result of the initially subordinate males becoming more dominant. If they were, then they should be less suppressed by more dominant males, and thus should have increased the amount of male-directed song they sang. We compared the change in overall amount of male-directed song sung from sham introductions and lesion introductions for males above the median dominance rank and males below median dominance rank. Lower ranking males did sing more during the lesion introductions (mean change in songs sung  = +10.33+ 39.17), whereas high ranking males slightly decreased their male directed song (mean change in songs sung  = −25.8±29.36), but owing to the substantial variability, this difference did not reach statistical significance (t(10) = .74, p>.46). Thus while the introduction of the lesioned females disrupted the typically stable singing patterns of males, we could find no consistent patterns of change in the males' dominance relationships.

Lesioned females may have caused this disruption of the male dominance hierarchy by providing lower-ranked males with opportunities to sing to lesioned females. There was a strong negative relationship between males' initial dominance rank and the amount they sang to lesioned females (spearman r = −.903, n = 12, p<.0001). Subordinate males (those males below median dominance rank) significantly increased the proportion of their song directed to lesioned females post-lesion (t(5) = 2.71, p<.05). Whereas, dominant males (males above median dominance rank) did not change the proportion of song they directed to lesioned females post-lesion (paired t-test, t(5) = 0.57, p>.52). These patterns reveal that sham-lesioned females pair-bonded with dominant (high quality) males whereas lesioned females did not show such selectivity.

We used social network metrics to compare group structure during the two rounds of introductions. Past work has suggested that groups characterized by stable, interactive singing networks have higher levels of reproductive success [5], [8], [24]. A variety of measures suggested that network stability and interactivity were reduced when lesioned females were added. While indegree and outdegree centrality (measures of network activity that characterize overall amounts of singing interactions occurring among males) did not change significantly when lesioned females were introduced (both ts(11) <0.35, both NS), we found that proximity (distance of a male to all others in the network) decreased (t(11) =  7.62, p<.0001), ego network size (the number of other individuals with whom each male sings) decreased (t(11) = 3.52, p<.005) and the number of weak, or indirect connections within groups increased (t(11) = 2.28, p<.017).

**Figure 4:**
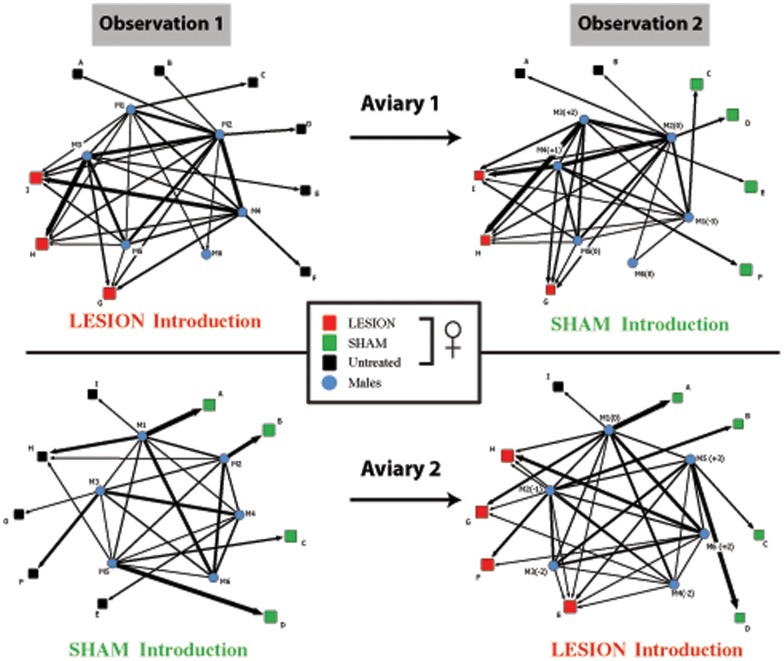
Lesioned females destabilize the social network of mixed-sex flocks. Social network structure for each observation session in experiment 2. Lines represent directed singing interactions. Letters/numbers beside nodes represent individual identity, (females: squares males: circles). Vertical spacing of males represents relative dominance rank based on male-directed singing ratio. Numbers beside males in parentheses represent change in dominance rank from the observation session before the one shown. Thickness of the line represents number of songs sung (largest line  = 208 songs [aviary 2, lesioned introduction, M1 to A], smallest  = 5 songs [multiple instances]). Arrows represent direction of singing interaction.

Thus, after introduction of lesioned females males changed position in the social network and changed how they interacted with others. A visualization of the difference in network structure when lesioned and sham-lesioned females were introduced to the groups is provided in Figure 4.

## Discussion

The effects of lesions targeted to HVC – examined at the level of the individual and also of the group – provided a means to understand the mechanisms and function underlying sexual preferences in female cowbirds. It also provided new insights into the role individuals can play in structuring social environments.

### Individual behavior

Selectivity in responding to variants of male song is a consistent and reliable feature of female cowbird copulation solicitation display behavior. Given any set of cowbird songs used in a playback session, some songs in the set will elicit more solicitation displays than others (see [21] and the pattern by sham-lesioned females in Figure 2). Lesioning HVC and the area surrounding it eliminated this selectivity. Lesioned females responded vigorously to all cowbird songs, though this enhanced tendency to respond to songs was for conspecific song only. Lesioned females did not respond to playbacks of heterospecific song.

The elimination of selectivity as a result of HVC-targeted lesions suggests that HVC and surrounding auditory areas in female cowbirds might play an important role in linking salient features of sexually-stimulating songs with the decisions of whether or not to elicit a postural response. The ability of HVC lesions to eliminate response preferences for high quality conspecific songs fits well with lesion studies in canaries [11] as well as with observations that HVC neurons can discriminate between sexually stimulating and non-stimulating songs [17]. In males, HVC lies at the junction between the auditory and the vocal motor system and while much is known about its role in song production [26], [27], its role in postural control, which is often associated with song in many species, has not been characterized. Interestingly, posture display during singing in male cowbirds – and probably many other species – is associated with changes in respiratory drive [28], which are thought to be influenced by HVC and its downstream target RA (Robust nucleus of the Archipallium) [29], [30]. Preliminary observations that HVC premotor activity might be correlated with these changes in posture-induced respiratory drive (Cooper & Goller, personal communication) suggest that HVC might in fact play a critical role in posture control in both males and females.

### Social behavior

Experimentally disrupting the sexual preferences of a subset of females in a group provided a means to study how social behaviour among group-mates is interconnected and dynamically responsive to social change. When lesioned females were introduced to mixed-sex flocks of freely behaving birds, they caused a cascade of effects throughout the social network, resulting in instabilities of the social system that disrupted courtship and competition of all the group-members.

The group-level results were all the more surprising given that – based on the behaviors we could measure (approaching males, producing chatter, allowing males to sing to them) – we could detect remarkably few specific behaviors that distinguished the lesioned females from the sham-lesioned females (other than patterns of chattering, see below). It was only when we examined the social behaviour among the group mates that we observed the pronounced effects of the introductions of the lesioned females. Thus it was the behaviour of the other birds that provided an assay to examine how the lesioned females' behaviour differed.

Often in songbirds, the overt behaviors of males become the focus of research attention while the females' subtle suite of behaviors and their role in influencing group dynamics get overlooked. Lesioned females had effects on aspects of behavior that have traditionally been considered to be under the control of males. For example, the introduction of the lesioned females influenced the male dominance hierarchy, and it influenced males' courtship patterns. While pair-bonding, mate guarding and singing selectivity all seem to be under the control of males, the pronounced differences seen in how males courted sham and lesioned females (Figure 3) also indicates that females have some control over these variables. While females have been implicated to play a role in male competition and male courtship in numerous species [31], [32], [33], it is rarely the case that the relationships between the sexes can be experimentally manipulated as was done here.

The one behavior that did differentiate the females by condition was in their chattering selectivity and it might have contributed to the behavioral mechanism that led to the cascade of effects after the introductions. While sham females chattered in response to their pair-mate, lesioned females were less selective in chattering, giving chatter calls in response to multiple males. Chatter can motivate males to pursue and court females, thus this lack of selectivity may have been an important factor in leading multiple males to court the lesioned females. Multiple males courting the same female can lead to aggressive interactions and disruptions to an otherwise stable male dominance hierarchy. The other females in the groups also reacted to the introduction of the lesioned females (and presumably to the change in activity of the males) by increasing the amount of undirected chatter to compete with the new females that were attracting so much male attention.

It is also possible, however, that females differed in a whole suite of subtle behaviors that influenced how males approached and sang to them. Males will only sing to females at very close distances (typically within 30cm) and females can exercise their preference in who sings to them by regulating their social distance, thereby providing or removing opportunities for males to sing. While there was no difference in the rate in which females in either group approached males, it could be that the females might have been less likely to move away from males, or more likely to give males other indications of their preferences (e.g., wingstrokes, [34]) that were not visible to the observers.

This study highlights the interconnected nature of the individual and its social environment; where neural circuits organizing individuals' behavior are intricately linked together in order to produce species-typical social behaviors. These types of socially distributed systems demand a new way to study both the mechanisms and the evolution of individual behaviour. Only by examining brains in social contexts can links between neurophysiology and the evolution of a social system be realized.
